# Development and experience-dependent modulation of the defensive behaviors of mice to visual threats

**DOI:** 10.1186/s12576-022-00831-7

**Published:** 2022-03-07

**Authors:** Madoka Narushima, Masakazu Agetsuma, Junichi Nabekura

**Affiliations:** Division of Homeostatic Development, National Institute for Physiological Sciences, 38 Nishigonaka Myodaiji, Okazaki, Aichi 444-8585 Japan

**Keywords:** Defensive behavior, Development, Superior colliculus, Experience, Looming stimulus

## Abstract

Rodents demonstrate defensive behaviors such as fleeing or freezing upon recognizing a looming shadow above them. Although individuals’ experiences in their habitat can modulate the defensive behavior phenotype, the effects of systematically manipulating the individual’s visual experience on vision-guided defensive behaviors have not been studied. We aimed to describe the developmental process of defensive behaviors in response to visual threats and the effects of visual deprivation. We found that the probability of escape response occurrence increased 3 weeks postnatally, and then stabilized. When visual experience was perturbed by dark rearing from postnatal day (P) 21 for a week, the developmental increase in escape probability was clearly suppressed, while the freezing probability increased. Intriguingly, exposure to the looming stimuli at P28 reversed the suppression of escape response development at P35. These results clearly indicate that the development of defensive behaviors in response to looming stimuli is affected by an individual’s sensory experience.

## Background

Organisms exhibit specific behavioral patterns in response to aversive stimuli to protect themselves and survive [1, 2]. Although species-specific patterns of defensive behaviors are genetically encoded, individuals’ experiences gained from their habitats affect the characteristics of these defensive behaviors [3, 4]. In particular, sensory experiences of the same modality as the triggering stimulus are expected to impact post-maturity behaviors. To understand the influence of an individual’s sensory experience on innate defensive behavior, the developmental process of the behavior must be described, and the developmental plasticity of the sensory system required to receive the aversive stimuli must be considered.

Rodents exhibit defensive behaviors, such as rapid escape or sudden freezing, when they recognize looming shadows above them [5, 6]. The defensive response to a visual threat is an ideal model for studying the effects of sensory experience during the development of post-maturity behaviors as it is solely triggered by the visual sense through the activation of superior collicular neurons [7, 8], and an individual’s visual experience can be easily manipulated [9]. It is generally accepted that an individual’s experience in its habitat can modulate the phenotype of defensive behavior. For example, wild mice, laboratory mice, or laboratory mice reared in different animal facilities have distinct characteristics or proportions of fleeing or freezing in response to the same threat stimulus [3, 5]. However, the effects of the systematic manipulation of visual experiences on vision-guided defensive behaviors have not been studied. Therefore, in the present study, we aimed to describe the developmental process of defensive behaviors in response to looming shadow stimuli and the effects of visual deprivation during development. We found that the probability of exhibiting an escape response, a typical defensive behavior, increased after postnatal day (P) 21, peaked at P28, and then stabilized. The probability of exhibiting the freezing response did not change significantly during development in our colony. When the visual experience was perturbed by rearing in a dark box from P21 for a week, the probability of the escape response decreased markedly, whereas the freezing probability increased. Intriguingly, exposure to looming stimuli at P28 could reverse the suppression of escape probability at P35. These results clearly indicate that the development of defensive behaviors toward looming stimuli is affected by the individuals’ sensory experience.

## Methods

### Animals

C57BL/6JJmsSlc mice aged between P16 and P56 were used. Mice were brought to our animal facility 1 week prior to the behavioral experiments and reared with their mothers until P28. At least two mice lived together after weaning. Mice were kept in a room with a 12-h light/dark cycle (7 a.m. on/7 p.m. off) and a temperature of 23 ± 1 °C. The animals were allowed to forage freely for food and water throughout the day. Cages were cleaned once every 2 weeks before 2 weeks of age, and once a week thereafter.

### Behavioral tests

**Fig. 1:**
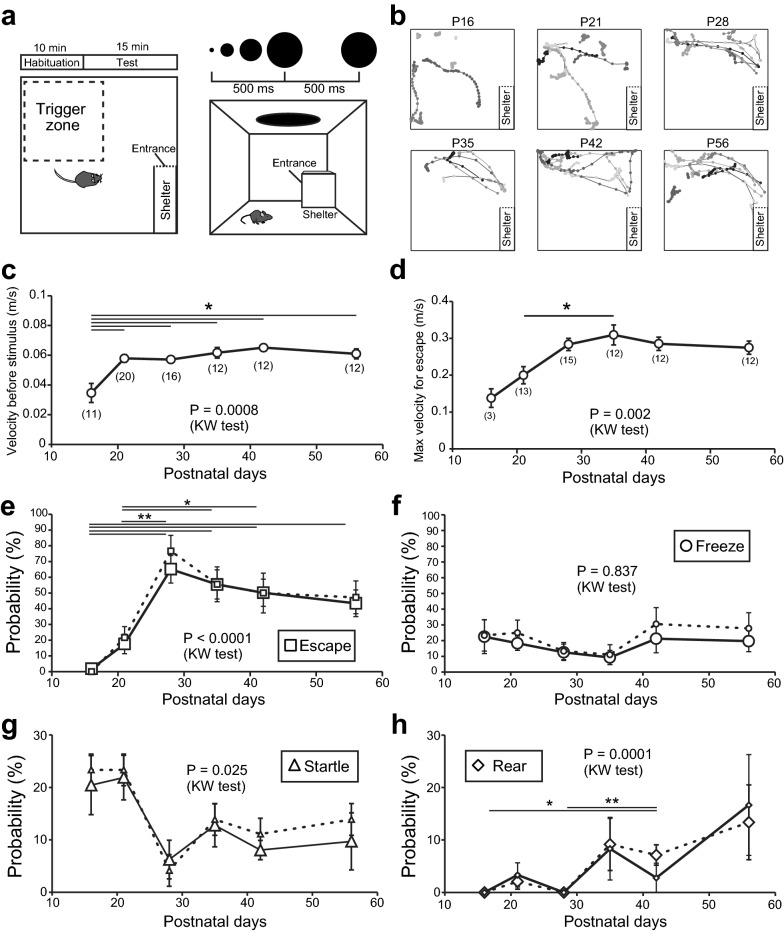
Development of defensive behaviors to visual threat. **a** Schemata of the behavioral test. An entrance to the shelter is represented by a dotted line. **b** Trajectories of mice after expanding disk stimuli were applied. Traces obtained from a single naïve mouse of the indicated age are shown in each panel. **c** Plots for the development of average velocity during the 10 s before the onset of visual stimuli. **d** Plots for the development of maximum velocity for escape. The maximum velocity during the 5 s after the onset of visual stimuli was collected. **e**–**h** Developmental change in probabilities of escape (**e**), freezing (**f**), startle (**g**) and rearing (**h**) response to expanding disk stimuli. Smaller symbols connected with dotted lines represent the average probability calculated from the first three trials during the 15-min test period. * or ** represents *P* < 0.05 or *P* < 0.01 with Steel–Dwass test following Kruskal–Wallis (KW) test. Error bars, ± SEM

All mice used for development analysis were naïve to the visual looming stimulus. We used equal numbers of females and males, except in the case of death just before the behavioral test, and mixed the data of both sexes for the analysis. The behavioral experiments were conducted during the light cycle between 11:00 a.m. and 5:00 p.m. On the day of the behavioral test, before the test period started, they were left in the test field (width 35 cm, length 35 cm, height 30 cm) with a shelter (width 5 cm, length 15 cm, height 10 cm) that had an entrance opened on the short side for 10 min for habituation with no stimulation (Fig. 1a). During the 15-min test period, visual stimulation was manually replayed on a monitor that covered the top of the test field when the mice entered a quarter of the test field, opposite to the other quarter that included the shelter (Fig. 1a). Visual stimulation was performed at 1-min intervals if the mouse entered the trigger zone repetitively or remained immobile in the trigger zone. The visual looming stimulus was a black disk that expanded from 0.2° to 40° of the mice’s visual angle in 500 ms and remained at a consistent size for 500 ms. The stimulus was repeated three times at 500 ms intervals (Fig. 1a). The production and application of visual stimulation, and control of recording using a GigE camera (ace acA 1300, Basler AG, Ahrensburg, Germany) were performed using custom-made software (Lab Squirrel, Australia). The behavior of the mice were recorded at 30fps.

### Analysis

The tracking software (ANY-maze, Stoelting Co., IL, USA) was used to convert the location of the mice from the recorded movies to numerical coordinates. Behavioral indicators, such as the timing of entering and leaving the shelter, duration of the time spent in the field or in the shelter, locomotion speeds, or duration of time spent immobile, were calculated using ANY-maze or MATLAB software (MathWorks, MA, USA).

We termed escape response as the mouse running into the shelter within 5 s of stimulus onset at a maximum speed three times faster than the mouse’s average speed over 10 s before stimulation, and freezing response as 85% of the mouse’s body area remaining immobile for more than 2 s. If the mouse ran as fast as the escape response but did not enter the shelter, the response was defined as a startle. The rearing response was manually counted if the mouse stood on its hind limbs during 5 s after the onset of stimulation.

**Fig. 2:**
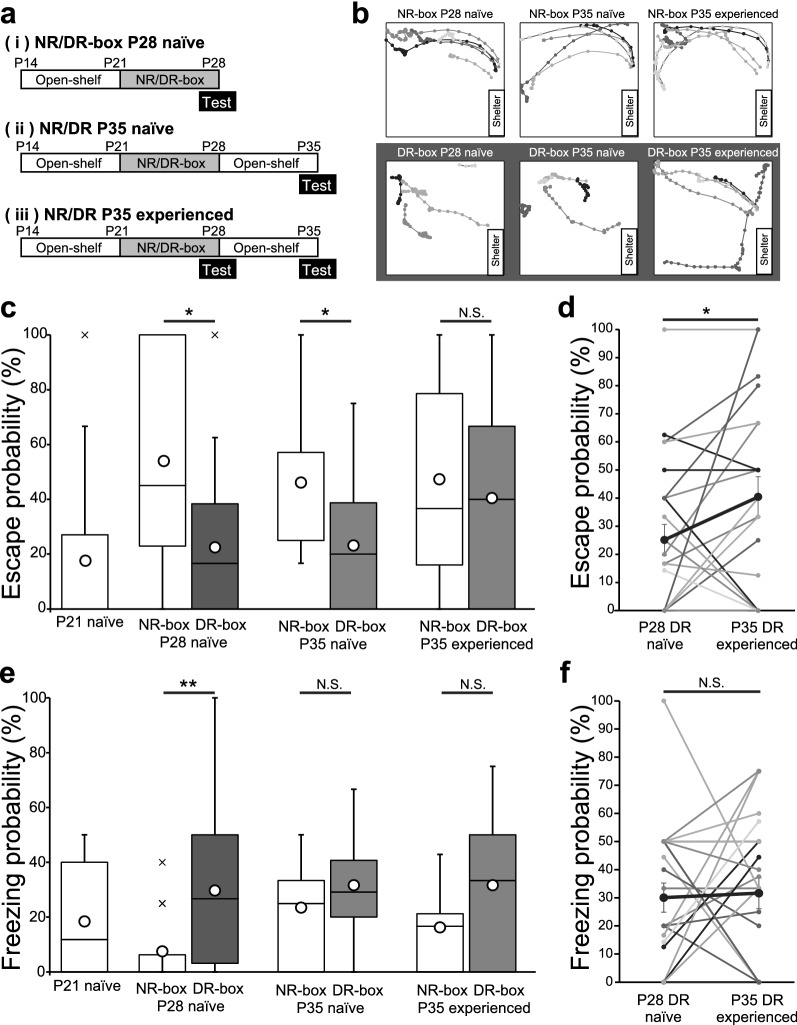
Visual experience modulates defensive behaviors to visual threat. **a** Time course for visual experience modification. (i) Mice spent a week in an open-shelf before they were put in an isolation box with (NR-box) or without (DR-box) lighting at P21. They spent a week in the box and behavioral tests were performed at P28 (NR-/DR-box P28 naïve). (ii) After a week of NR- or DR-box rearing, mice were returned to an open-shelf condition for an additional week before the behavioral test was performed at P35 (NR-/DR-box P35 naïve). (iii) Mice used for NR-/DR-box P28 naïve condition (i) were returned to the open-shelf, then tested again at P35 (NR-/DR-box P35 experienced). **b** Representative trajectories of NR-/DR-box mice after indicated rearing conditions in response to expanding disk stimuli. **c** and **e** Box plots representing probabilities for escape (**c**) and freezing (**e**) responses to expanding disk stimuli after the indicated rearing conditions. A box represents the first and third quartiles. The whiskers represent the sample minimum and maximum, respectively. White dot, mean; line in the box, median; cross, outlier. * represents *P* < 0.05 with the Mann–Whitney *U* test. N.S., not significant. **d** and **f** Comparison of escape (**d**) or freezing (**f**) probability between the same DR-box P28 naïve and DR-box P35 experienced mice. Gray dots connected with a line represent data obtained from the same mouse at P28 and P35. Black dots indicate the mean value. Error bars, ± SEM. * represents *P* < 0.05 with the Wilcoxon signed-rank test. N.S., not significant

The probability of each behavioral response was calculated for each mouse by dividing the number of times each behavioral response was recorded by the number of stimulation trials given during the 15-min test period, and the average was calculated for each age group. We also calculated the response probability for the first three trials in the developmental analysis. In the box plots in Fig. 2, the upper and the lower whiskers represent 1.5 times the first or the third interquartile range (IQR). Data points more than 1.5 times the IQR above the upper quartile and below the lower quartile (Q1 − 1.5 × IQR or Q3 + 1.5 × IQR) were defined as outliers. The Kruskal–Wallis test and multiple comparisons by the Steel–Dwass test were used to compare changes due to development. The Mann–Whitney *U* test or Wilcoxon signed-rank test was used for comparisons between the two groups.

### Modulation of visual experience

To modulate the visual experience, a mother and children aged P21 in a breeding cage were placed in an isolation box with LED lighting and a ventilation fan (ISB-1, Bio Research Center Co., Ltd., Aichi, Japan) for a week. The lighting was turned on and off every 12 h for normally reared mice and kept off for dark-reared mice. All were naïve to the visual looming stimuli on the day of the behavioral test at P28. Some mice underwent a second behavioral test a week after the initial test at P35.

## Results

### Developmental time course of defensive behaviors to visual threats

First, we investigated how defensive behaviors against visual threats develop in mice. We tested the behavioral responses to looming stimuli in mice aged between P16 and P56. At P16, only a few days after eye-opening, mice slowly explored the test field or stayed at the position at which they were placed for the entire habituation and test periods. The locomotion speed before stimulation was the slowest (0.035 ± 0.006 m/s, *N* = 12 mice) among the mice of all ages (Fig. 1c); therefore, normal locomotion appeared immature. In parallel with this result, most (11 out of 12 mice) P16 mice did not escape the looming stimuli (Fig. 1b, e). The most frequently observed case was no behavioral response to the stimulus (36.0 ± 10.9%). However, P16 mice seemed to recognize the stimulus, because they froze during and after stimulation (4 mice, 22.5 ± 10.7%) (Fig. 1f), though not every time, or they showed a startle-like sudden acceleration of locomotion speed during stimulation (7 mice, 20.4 ± 5.6%) (Fig. 1g). They also seemed to recognize the presence of the shelter, because 8 out of the 12 mice entered the shelter more than once (6.3 ± 1.1 times) (Table 1), except for 4 that entered the shelter during the habituation period and never went out during the entire test period. The speed of startle-like behavior reached speeds of > 5 times faster than their normal locomotion speed (0.19 ± 0.02 m/s, 7 mice), but they rarely went to their shelter after the stimulation.

**Table 1:** Comparison of behavioral features during the test period among developmental ages

Condition	Shelter entry except for escape***	No. of trials ***	Time in the field (s)**	Time in the shelter (s)***
P16 (N = 11)	6.3 ± 1.1	4.1 ± 0.8	364.0 ± 80.8	530.6 ± 82.7
P21 (N = 20)	5.8 ± 0.6	6.0 ± 0.4	501.6 ± 44.9	381.6 ± 44.6
P28 (N = 16)	6.7 ± 1.1	5.1 ± 0.4	264.7 ± 36.2^‡^	611.0 ± 33.8^‡^
P35 (N = 12)	10.4 ± 1.4^‡,§§^	8.0 ± 0.4^†,§§^	483.7 ± 31.3^§^	326.7 ± 31.7^§§^
P42 (N = 12)	12.6 ± 1.9^‡‡,§§^	8.4 ± 0.6^†,‡,§§^	447.2 ± 38.7	336.3 ± 40.4^§§^
P56 (N = 12)	11.4 ± 1.1^‡‡,§§^	8.0 ± 0.4^†,§§^	410.0 ± 18.5	353.8 ± 24.7^§§^

Behaviors during the test period were compared among developmental ages. The number of times the mice entered the shelter and the trigger zone tended to increase with development. P28 mice stayed in the shelter significantly longer than other age groups. ** or *** indicates P < 0.01 or P < 0.001 with the Kruskal–Wallis test. †, ‡ or § indicates P < 0.05 compared to P16, P21, or P28 data with multiple comparisons of the Steel–Dwass test performed after the Kruskal–Wallis test. Two or three symbols indicate P < 0.01 or P < 0.001, respectively

At P21, the locomotion speed of the mice reached 0.058 ± 0.002 m/s (*N* = 20 mice), which was as fast as that of the adult mice (P56, 0.061 ± 0.003 m/s, *N* = 20 mice) (Fig. 1c). Thirteen of the twenty P21 mice exhibited escape responses to the looming stimulus (Fig. 1b, e). The occurrence probability of escape behavior was higher than that of P16 mice, but it still made up < 20% of the response (17.6 ± 6.2%) (Fig. 1e). The maximum escape speed was 0.20 ± 0.02 m/s which was as fast as that of the P16 startle-like response (Fig. 1d). Similar to P16 mice, P21 mice exhibited freezing (18.3 ± 4.5%) or startle-like (21.9 ± 4.3%) behaviors in response to the looming stimulus as frequently as escape behavior. In contrast, the probability of no behavioral response was reduced to 10.4 ± 2.4%.

The escape probability increased suddenly at P28 and reached > 60% (65.8 ± 8.8%) (Fig. 1b, e). Despite the normal locomotion speed not differing significantly from that of P21 mice (0.058 ± 0.002 m/s, *N* = 16 mice; *P* = 0.41 compared to P21 mice with Mann Whitney *U* test) (Fig. 1c), the maximum speed at which they escaped was significantly faster than observed in P21 mice (0.28 ± 0.02 m/s, *P* = 0.004) (Fig. 1d). In parallel with the increase in escape probability, the occurrence of startle-like responses decreased to 6.3 ± 3.7%, which was significantly lower than that in P16 or P21 mice (*P* = 0.004 with Mann–Whitney *U* test) (Fig. 1g). The probability of freezing behavior was slightly reduced (11.5 ± 5.3%) but did not differ significantly from that of P21 (*P* = 0.167) (Fig. 1f). This suggests that startle-like behavior at immature ages may be substituted by escape behavior at P28.

The escape probability of mice aged around P35–56 stayed at around 50% (at P35, 54.3 ± 8.9%; at P42, 50.1 ± 8.6%; at P56, 43.4 ± 8.5%; *N* = 12 mice, respectively) (Fig. 1b, e). In contrast to the dramatic change in escape probability, the freezing probability did not change significantly throughout development (P35, 7.3 ± 4.6%; P42, 19.4 ± 8.3%; P56, 15.1 ± 5.3%) (Fig. 1f). The probability of startle-like behavior remained at around 10% after it lessened at P28 (at P35, 12.8 ± 4.1%; at P42, 8.1 ± 1.8%; at P56, 9.7 ± 5.4%) (Fig. 1g). It is noteworthy that other types of alert responses, such as rearing, have emerged along with development. A rearing response was observed at P21. Its incidence then increased to approximately 10% of the responses to looming stimuli (P35, 9.2 ± 5.0%; P42, 7.1 ± 1.9%; P56, 13.4 ± 7.1%) (Fig. 1h).

We fixed the test duration at 15 min and analyzed the responses to the visual threat given during the period of spontaneous exploratory behavior. However, mice at P28 or younger tended to enter the trigger zone less frequently than older mice, and other features of behaviors, such as shelter entry, or time spent in the field or shelter were also different among age groups (Table 1). Because older mice tended to experience more trials than P28 or younger mice, the developmental changes in the response probabilities might have resulted from habituation to the visual stimuli during the test session. Therefore, we calculated the response probability from the first three trials of the test period (smaller symbols are connected with dotted lines in Fig. 1e–h). We did not find a clear difference in the probability that was calculated from the first three trials or all trials; therefore, habituation would not contribute to the developmental changes in the response probabilities.

In summary, escape behavior developed gradually with the highest probability at P28, whereas freezing probability remained unchanged throughout development. The responses became more variable with development, suggesting that mice acquired the ability to choose appropriate behavior for each situation.

### Visual experience modulates development of defensive behaviors to visual threats

Because the escape probability dramatically increased during the period between P21 and P28, at the time when visual experiences affect vision-related neuronal circuits [10–12], we hypothesized that visual experience affected the phenotype of defensive behaviors. To test this, we reared the mice in isolation boxes with a normal 12 h light/dark cycle (normally reared; NR-box) or without any lighting (dark reared; DR-box) from P21 for a week before testing their responses to the visual threat (Fig. 2a). The boxes containing both mouse groups were closed for a week until the mice were removed at P28 for behavioral tests. First, we analyzed their behavior during habituation (Table 2) and the test period (Table 3) to test whether the behavior of the mice was affected by dark rearing, because their circadian rhythms might change. Although a slight difference was observed in the time spent mobile in the field during the habituation period (DR, 231.7 ± 25.6 s vs. NR, 344.4 ± 32.1 s; *P* = 0.045 with Mann–Whitney *U* test), there was no difference in other features of behavior or the number of trigger zone entries. These results suggest that the effects of dark rearing on circadian rhythms are likely to be minor. The normal locomotion speed (*P* = 0.98) or the maximum speed at escape (*P* = 0.42) did not differ between the groups that experienced different lighting conditions (Tables 3 and 4), indicating that locomotor ability developed normally even without visual experience for a week.

**Table 2:** Comparison of behavioral features during the habituation period between rearing conditions

Condition	Speed (m/s)	Travel distance (m)	Time mobile in the field (s)	Time immobile in the field (s)	Time in the shelter (s)
NR-box P28 naive	0.026 ± 0.005	11.3 ± 2.2	344.4 ± 32.1	101.5 ± 47.4	155.8 ± 39.3
DR-box P28 naive	0.018 ± 0.002	6.3 ± 0.6	231.7 ± 25.6*	138.7 ± 34.7	222.4 ± 60.3

Behaviors during the habituation period which was just after the mice were taken out from the rearing box were compared. * indicates P < 0.05 with the Mann–Whitney *U* test

**Table 3:** Comparison of behavioral features during the test period between rearing conditions

Condition	Speed (m/s)	Time in the field (s)	Time in the shelter (s)	Shelter entry except for escape
NR-box P28 naive	0.056 ± 0.004	297.1 ± 59.8	571.1 ± 65.7	6.9 ± 1.3
DR-box P28 naive	0.053 ± 0.002	432.2 ± 35.1	394.1 ± 37.8	6.6 ± 0.7
NR-box P35 naive	0.047 ± 0.002	513.8 ± 47.4	334.5 ± 40.3	7.3 ± 0.9
DR-box P35 naive	0.052 ± 0.001	455.7 ± 40.6	405.8 ± 39.4	10.2 ± 1.1
NR-box P35 experienced	0.054 ± 0.003	361.6 ± 37.9	459.6 ± 44.8	8.5 ± 0.9
DR-box P35 experienced	0.046 ± 0.002	458.1 ± 38.2	404.5 ± 36.3	9.1 ± 0.8

Behaviors during the test period were compared between rearing conditions for each age. The speed before the onset of expanding disk stimulation, duration of the time spent in the field or in the shelter, or number of shelter entries except for escape did not differ significantly. *NR* normally reared, *DR* dark reared

**Table 4:** Comparison of other features of defensive responses between rearing conditions

Condition	No. of trials	Max speed for escape (m/s)	Startle probability (%)	Rear probability (%)
NR-box P28 naive	5.0 ± 0.7	0.254 ± 0.033	13.0 ± 6.0	2.1 ± 2.1
DR-box P28 naive	5.0 ± 0.3	0.267 ± 0.019	21.3 ± 4.0	3.2 ± 1.5
NR-box P35 naive	5.4 ± 0.4	0.290 ± 0.026	13.5 ± 3.5	5.8 ± 3.1
DR-box P35 naive	5.3 ± 0.3	0.266 ± 0.017	16.9 ± 4.3	1.3 ± 0.9
NR-box P35 experienced	5.8 ± 0.3	0.299 ± 0.036	16.1 ± 4.2	4.2 ± 2.2
DR-box P35 experienced	5.7 ± 0.4	0.295 ± 0.022	21.6 ± 4.3	9.3 ± 0.1

Features of defensive behaviors other than the escape and freezing probabilities were compared between rearing conditions. The number of trials, maximum speed when mice escaped, or probabilities of startle or rearing responses did not differ significantly. *NR* normally reared, *DR* dark reared

Next, we analyzed the responses of NR- or DR-box mice to visual threats. Interestingly, mice reared in the DR-box from P21 for 1 week exhibited a marked reduction in escape probability (22.3 ± 4.7%, *N* = 30) compared to mice reared in the NR-box (54.0 ± 11.4%, *N* = 12, *P* = 0.014, Mann–Whitney *U* test) (Fig. 2b, c). In contrast, the freezing probability of DR-box mice (29.7 ± 4.6%) was significantly higher than that of NR-box mice (7.5 ± 5.1%, *P* = 0.005) (Fig. 2b, e). The probability of escape or freezing in DR-box mice was not different from that of P21 naïve mice (escape, *P* = 0.96; freezing, *P* = 0.12 with Mann–Whitney *U* test) (Fig. 2c). The duration that they spent in the field or shelter also did not differ significantly between rearing conditions, (field, *P* = 0.18; shelter, *P* = 0.06, Mann–Whitney *U* test) (Table 3), and both groups of mice approached the shelter with similar frequency between stimuli (*P* = 0.81, Mann–Whitney *U* test) (Table 3). These facts indicate that DR-box mice could recognize the shelter; therefore, the reduced probability of escape was not linked to them not finding the shelter. The probabilities for other types of behavioral responses to visual threats such as startle-like (13.0 ± 6.0% for NR-box vs. 21.3 ± 4.0% for DR-box, *P* = 0.13) or rearing behaviors (2.1 ± 2.1% for NR-box vs. 3.2 ± 1.5% for DR-box, *P* = 0.15) were not different between mice rearing conditions (Table 4). These results suggest that mice could recognize visual threats, but experiencing a week of darkness changed their decision to escape or freeze.

We then wondered whether such experience-dependent changes in defensive behavior preference could be reversed by visual experience. We prepared another group of naïve mice that were reared in NR- or DR-box conditions from P21 for a week, returned to an open shelf, and left for an additional week (Fig. 2a). The escape probability of P35 mice that experienced a week of DR-box 1 week prior was as low as that of mice at P28 just after being removed from the dark box (23.1 ± 5.2%, *N* = 23 mice, *P* = 0.933) and significantly lower than that of mice reared in the NR box from P21 which were then left on an open shelf for a week (42.0 ± 7.6%, *N* = 12 mice, *P* = 0.033) (Fig. 2b, c). Similarly, the freezing probability of these mice remained high (31.7 ± 3.7%, *P* = 0.648 compared to P28 DR-box) (Fig. 2b, e). The results indicated that a week of visual experience in the open shelf was not sufficient to rescue the maturation of escape behavior.

Interestingly, however, when the mice had experienced a visual threat once at P28 after dark rearing (Fig. 2a), the escape probability at P35 nearly doubled after they spent a week in the open-shelf (40.5 ± 7.2%, *N* = 24, *P* = 0.046, Wilcoxon signed-rank test) (Fig. 2d). This suggests that experiencing a visual threat rather than a daily visual experience contributes to the development of escape behavior. Conversely, the probability of freezing in P35 mice remained as high as in P28 mice just after dark rearing (31.6 ± 5.5%, *N* = 24, *P* = 0.965, Wilcoxon signed-rank test) (Fig. 2f), indicating that the increased tendency to choose freezing behavior caused by dark rearing was irreversible, even after a week of normal or frightening visual experiences. These findings clearly indicate that although the effect of dark rearing on escape or freezing behaviors remained even after a week of normal visual experience, escape behavior can still develop following exposure to a visual threat.

## Discussion

Wild mice often roam away from their homes to gather food, regardless of safety. Therefore, weanling mice need to acquire the ability to flee or freeze in response to aversive stimuli for survival during development. In the current study, we clarified the developmental process of defensive behaviors in mice in response to visual looming stimuli. Mice opened their eyes around the second week postnatally and weaned by the fourth postnatal week. During 2 weeks, their motor abilities matured and mice began exploring voluntarily. The visual system also matures [9], including retinal inputs to the superior colliculus [13, 14], a brain region that triggers defensive behaviors against visual threats [7, 8]. At P16 (before weaning), the mice did not escape to the shelter, but froze or exhibited a startle-like response with short-distance rapid running. This suggests that they could recognize an approaching object, but did not escape. The speed of voluntary exploration was slow in P16 mice; therefore, it would be safer to stay in the same place, where they recognized the stimuli than to run towards the shelter. Mice voluntarily explored the test field more often at P21, and their visual systems were more developed than those of P16 mice; however, the escape probability was still low. They responded with freezing or startle-like behavior with a probability similar to that of the P16 mice. Interestingly, escape behavior rapidly increased between P21 and P28, which is the same timing as the reduction of probability for a startle-like response and increment of the maximum speed for escape. Unlike a startle-like response, escape behavior requires not only visual input but also the perception of danger and learning the position of a hiding place [1, 15, 16]. The development of neural connectivity between brain areas responsible for these different functions in addition to the maturation of motor ability might be necessary before an escape response can be acquired.

The present study also showed a visual experience-dependent modification of defensive behaviors after a week of visual deprivation (Fig. 2). Modulating visual experience results in plastic changes in various synapses in the visual system [9], but the direct effect on vison-triggered behaviors is less understood. Because stress can enhance escape behavior [17, 18], we chose dark rearing, which did not harm the mice’s bodies, instead of other ways of visual deprivation, such as eyelid sutures. Intriguingly, a week of dark rearing from P21 markedly reduced the probability of an escape response and increased the probability of a freezing response compared to mice reared in a box with normal lighting. The direction of change was opposite to that caused by stress [17, 18]; therefore, mechanisms other than stress could underlie behavioral plasticity. The free-running period of mice is slightly shorter than 24 h so the activity cycle would shift for several hours toward earlier starting of the active period after 1 week of dark rearing [19]. Even if the cycle shifted, it is not expected to have a significant effect, because it has been reported that there is no difference in defensive behaviors during the light and the dark cycle [5]. In addition, we observed a decrease rather than an increase in the time spent active during the habituation period and the difference disappeared during the test period. Therefore, the change in the circadian rhythm would have a minor effect on the defensive behaviors. The same rearing strategy weakens synaptic strength and remodels the connection pattern in the retinogeniculate synapses [10–12], so the visual perception of DR-box mice at P28 and P35 would be lower than that of NR-box mice. However, the DR-box mice approached and stayed in the shelter as much as the NR-box mice and exhibited freezing or other types of responses to the stimuli, indicating that they perceived the surrounding environment and aversive visual stimuli. Because the superior colliculus is the other recipient nucleus of retinal inputs, similar plasticity may occur in the retinal synapses of collicular neurons that administer escape responses. Conversely, there should be mechanism(s) that increase freezing response after dark rearing. Although the same looming stimuli trigger two types of responses, distinct subclasses of neurons in the superior colliculus and different downstream pathways may cause these responses [7, 20–24]. How sensory experience affects neuronal circuits that balance the occurrence of escape or freezing behavior [21, 25–27] should be understood to unravel the neural basis for experience-dependent modification of defensive behaviors.

It was surprising that resumption of normal visual experience for a week did not promote the escape response, but exposure to looming stimuli at P28, in addition to normal visual experience, did (Fig. 2). After the mice were removed from the DR box, they were fed and cleaned for the same amount of time. Therefore, the specific experience of exposure to looming stimuli, rather than daily sensory information is necessary to link stimuli and escape behavior. The results of our study revealed that the escape response to visual threat is not an entirely ‘innate’ behavior, but that experience or learning, is necessary for its development. It is an intriguing subject for future studies to clarify, where the experience of visual threat is stored in the brain and how the brain region is connected to the neuronal circuits that administrate innate escape behavior. In contrast, the freezing response increased rather than decreased after DR exposure, and the probability of occurrence did not change after visual experience. This suggests that the development of neural circuits for freezing behavior is modulated by visual experience between P21 and P28, but in the opposite direction to the escape behavior. This difference in the developmental process of active and passive defensive behaviors may help researchers understand the locus of memory for visual threat experiences.

## Conclusion

Our experiments showed that manipulating visual experiences in early life influences innate vision-guided defensive behaviors. One week of deprivation of visual experience delayed the development of the escape response but increased the probability of the freezing response. Moreover, the development of escape response requires exposure to visual threats. Therefore, this system could be an appropriate model for understanding the brain mechanisms by which the growth environment influences the behavioral patterns of individual organisms. This system will soon allow us to directly link plasticity at the cellular level in the brain and a basis for behavioral selection that shapes an individual's unique personality.
